# Association between Tumor Size and Malignancy Risk in Hormonally Inactive Adrenal Incidentalomas

**DOI:** 10.7759/cureus.6574

**Published:** 2020-01-06

**Authors:** Veli Vural, Eyyüp M Kılınç, Demet Sarıdemir, İsmail B Gök, Amil Hüseynov, Alim Akbarov, Muhittin Yaprak

**Affiliations:** 1 General Surgery, Akdeniz University Hospital, Antalya, TUR; 2 General Surgery, Akdeniz University School of Medicine, Antalya, TUR

**Keywords:** adrenal incidentalomas, pheochromocytoma, adrenocortical cancer

## Abstract

Introduction

Adrenal incidentalomas (AI) are adrenal masses that are discovered during radiological examinations conducted for other reasons. In this study, we focused on the pathological and radiological properties of nonfunctional AI(NFAI) and the association with malignancy risk in our clinical series.

Methods

A total of 186 patients underwent adrenalectomy between 2010 and 2017; of these, 76 (40.8%) patients with non-functional AI were included in the current study. The radiological and pathologic characteristics of these AIs were retrospectively analyzed to determine the malignancy rate.

Results

There were 22 (28.9%) male and 54 (71.1%) female patients with nonfunctional AI included in this study. The median age was 55 (range: 24-85) years. Of the patients included, 37 (48.6%) had AI on the left and 39 (51.3%) had AI on the right adrenal gland. Sixty-one (80.2%) cases were treated laparoscopically, four (5.3%) required conversion to open surgery due to intraoperative difficulties such as bleeding and adhesions, and 11 (14.4%) were managed with open adrenalectomy. The rate of malignancy in the tumors with diameters of <4 cm, 4-6 cm, and >6 cm was found to be 0%, 2.9%, and 13.6%, respectively.

Conclusions

Determining the ideal cutoff value for surgical indication in an NFAI is challenging. Besides the malignancy risk, the rate of silent pheochromacytomas must be taken into account in the surgical decision.

## Introduction

An adrenal incidentaloma (AI) is described as “an adrenal mass that is serendipitously discovered by radiological examination intended for another reason”. First described more than three decades ago, AI has become a common clinical conundrum in terms of management [1-2]. It is important to recognize and treat AIs that have hormonal hyperactivity or a high risk of malignancy. Early-stage adrenocortical carcinoma (ACC) should always be considered a possibility after discovering an AI [3]. Approximately 5% of patients undergoing cross-sectional imaging have an adrenal mass, and of these, 5% are malignant [4]. In computed tomography (CT), a tissue density greater than 10 Hounsfield units (HU) may indicate malignancy and in CT studies with contrast, a washout rate lesser than 50% (i.e. clearance of contrast agent from the mass) strongly suggest malignancy. The evaluation of signal intensity on magnetic resonance imaging (MRI) scans may further facilitate the discrimination between benign and malignant lesions in certain cases [5]. Contrast washout on MRI provides similar information to that found on CT scans [6].

Two consensus statements have provided the algorithms for the treatment of AI: a consensus statement by the National Institutes of Health (NIH) in 2002 and by the American Association of Clinical Endocrinologists(AACE) / American Association of Endocrine Surgeons (AAES) in 2009 [7-8]. These statements recommend adrenalectomy for tumors that are hypersecretory, are >4-6 cm in size, or have suspicious radiological signs (e.g., irregular borders, heterogeneity, hemorrhage, central necrosis, or calcification) [3]. Despite these recommendations, there is a “gray zone” in the treatment of patients with high-density hormone-inactive tumors that are smaller than 4 cm and those with low-density hormone-inactive tumors between 4 and 6 cm but have no radiological sign suggesting malignancy in a CT scan.

Wang et al. reported that adrenalectomy is cost-effective in treating neoplasms >4 cm and when treating patients <65 years, primarily due to the aggressiveness of ACC [9].

We assessed the malignancy rate in non-functioning adrenal incidentalomas (NFAIs) and analyzed the factors associated with malignant pathological features.

## Materials and methods

Study design

**Figure 1:**
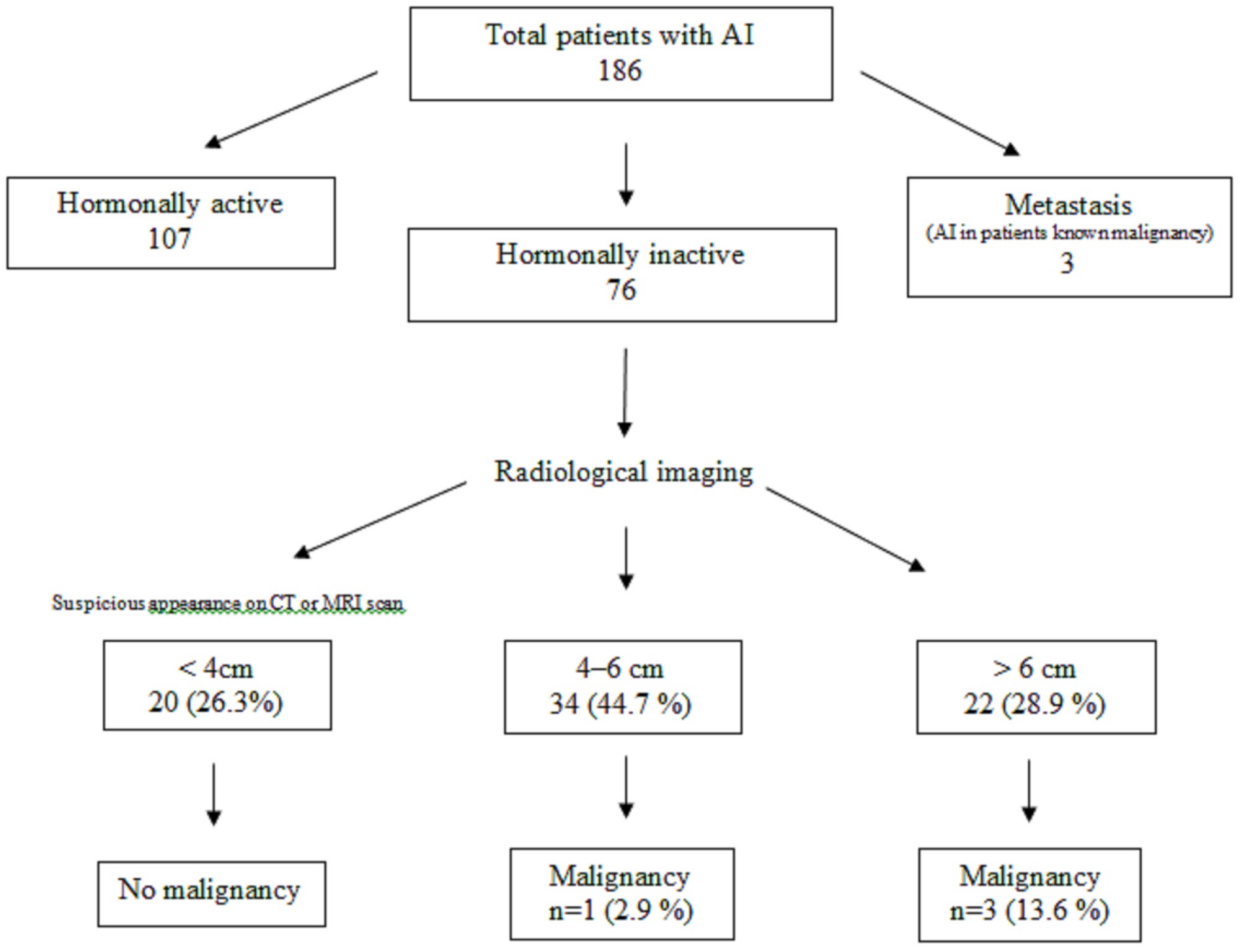
Flow chart of the study group based on AACE/AAES guidelines (i.e., the reasons for surgery in 76 patients with hormonally inactive AI) AI: adrenal Incidentaloma, CT: computed tomography, MRI: magnetic resonance imaging, AACE: American Association of Clinical Endocrinologists, AAES: American Association of Endocrine Surgeons

We performed a retrospective analysis of the patients who underwent adrenalectomy between 2010 and 2017 in our institution. The patients with hypersecretion-related symptoms and patients with known primary malignancy and suspected adrenal metastases were excluded from the study (Figure 1). 

All patients underwent a preoperative medical workup for adrenal hyperfunction. The baseline hormonal evaluation included the measurement of plasma cortisol, plasma aldosterone and plasma renin activity, serum dehydroepiandrosterone sulfate (DHEAS), salivary cortisol, and urinary epinephrine, norepinephrine, and cortisol assay. Additionally, serum potassium levels were monitored, and arterial blood pressure measurements were obtained in all patients. A final count of 76 patients was included in the study.

The reason for adrenalectomy for masses smaller than 4 cm was suspicious signs found during imaging, and for masses larger than 4 cm, it was the size of the mass.

This study was approved by the Ethics Committee of Akdeniz University.

Statistical analysis

Using the SPSS 16.0 software package (SPSS Inc., Chicago, IL), relationships between clinical features, treatment approaches, and pathological results (i.e., malignant or benign) were analyzed with the Chi-square test and Fisher’s exact test; a *p*-value lower than 0.05 was considered to be significant. Pearson’s correlation coefficient was used to analyze correlations between age and tumor size, an *r*-value greater than 0.5 or lesser than -0.5 was considered a strong correlation between the two.

## Results

Clinical characteristics of the patients

A total of 186 patients underwent adrenalectomy between 2010 and 2017 in our institution. Among these, 76 (40.8%) surgeries were for NFAI; 107 (57.5%) were for cases in which patients presented with symptoms related to hypersecretion and were then found to have adrenal pathology on imaging; and three (1.6%) were for cases in which adrenal metastasis from a known primary cancer was found during follow-up. In the group of 76 patients, 22 were male and 54 were female (71.1%). The median age was 55 (range: 24-85) years. 37 (48.6%) patients had a left-sided adenoma, while 39 (51.3%) had an AI on their right adrenal gland. In all, 61 (80.2%) cases were performed laparoscopically, four (5.3%) were converted to open surgery due to intraoperative difficulties such as bleeding and adhesions, and 11 (14.4%) were managed with open adrenalectomy.

Radiological evaluation

The radiological evaluation included CT scans in 57 patients, MRI scans in 15 patients, and ultrasonography (USG) in four patients. Radiological evaluation revealed 20 (26.3%) AIs with a diameter <4 cm, 34 (44.7%) with a diameter of 4-6 cm, and 22 (28.9%) with a diameter >6 cm (Table 1).

**Table 1:** Summary of the results of the radiological evaluations and pathologic outcomes of the 76 patients with hormonally inactive AI CT: computed tomography, MRI: magnetic resonance imaging, USG: ultrasonography

	CT (n = 57)		MRI (n = 15)		USG (n = 4)	
	Benign	Malignant	Benign	Malignant	Benign	Malignant
<4 cm	15	0	4	0	1	0
4–6 cm	25	1	5	0	3	0
>6 cm	14	2	5	1	0	0
Total	54	3	14	1	4	0

Pathological examination

The rate of malignancy in tumors with diameters of <4 cm, 4-6 cm, and >6 cm was found to be 0%, 2.9%, and 13.6%, respectively. When the cutoff point of 6 cm was defined, the tumors with a diameter of <6 cm had a significantly lesser incidence of cancer when compared to tumors with a diameter >6 cm (Table 1). The mean age of the four patients with malignancy was 57.25 (range: 45-73) years. The mean tumor size was 12.12 (range: 5.7-18) cm. Benign adrenal adenoma was the most frequent tumor with an incidence of 56.5% among our study group. The results of the pathological evaluation of 76 patients with NFAI are summarized (Table 2).

**Table 2:** Results of final pathological examination in 76 patients with hormonally inactive AI who underwent adrenalectomy AI: adrenal incidentaloma

Pathology	Number of patients	% of mean size
Benign adrenal cortical adenoma	43	56,5
Adrenocortical hyperplasia	9	11,8
Myelolipoma	8	10,5
Benign cyst	5	6,5
Pheochromocytoma	5	6,5
Hemangioma	1	1,3
Normal adrenal tissue	1	1,3
Adrenocortical carcinoma	3	3,9
Liposarcoma	1	1,3
Total	76	

## Discussion

In autopsy series, adrenal masses larger than 0.5 cm in size are rather common (occurring in approximately 2% to 9% of people), and adrenal masses larger than 1 cm in size are found in 1.3% to 3.4% of patients who undergo abdominal or chest CT scans [3]. Lou et al. reported that secondary imaging modality was performed in 98 of 264 (37.1%) cases, with MRI being the most commonly performed secondary imaging modality, used in 52 (53%) cases [10]. Unfortunately, studies for adrenal function are not useful in differentiating between benign and malignant tumors and no specific serum tumor markers are available.

Although there seems to be a consensus about the effectiveness of adrenalectomy for treating hyperfunctioning incidentalomas, there are differences in the published reports relating to the use of adrenalectomy for the treatment of NFAI [7]. In most studies, the size of the adrenal mass is considered an important criterion in the differentiation between benign and malignant masses, but the ideal cutoff value has yet to be defined. In our study, we found that malignancy rate was significantly higher in an AI with a diameter of >6 cm.

Hussain et al., using logistic regression analysis, estimated that the probability of malignancy ranges from 16% for a 2 cm mass to 82% for an 8 cm mass; however, Pantalone et al. reported that a growth cutoff value of 0.8 cm had the highest sum of sensitivity and specificity, of 72% and 81.1%, respectively [11-12]. Ballian et al. did not find any ACCs in lesions smaller than 4 cm and suggested that a 4 cm threshold for resection would identify primary malignant tumors and decrease the frequency of surgery being done for benign tumors; this threshold had a high sensitivity of 93% albeit a low specificity of 42% in predicting malignancy [13]. Moreover, in several series, most benign masses (76% to 100%) were larger than 5 cm in size [14-15].

In the literature currently available, different study groups offer similar indications relating to the use of adrenalectomy. In several recent series, the mean malignancy rate was reported to be 14.3% (range: 3.5% to 34%), possibly due to varying selection criteria among the patients [16].

Kutikov et al. reported their 22-year experience with adrenocortical cancer, in which 4,275 patients were analyzed, the median survival was 24 months, and localized adrenal cancer was detected in 43.9% of cases [17]. During the study period, there were no positive trends in terms of overall five-year survival rate which was disappointing as it was expected that the increase in radiographical screening would lead to diagnostic shifts over time, such as earlier detection and earlier stages and smaller sizes of tumors in the kidney and retroperitoneal organs at the time of detection. As a reason for this discrepancy, a more aggressive adrenal cancer growth pattern has been proposed.

The risk of adrenal cancer is associated with larger tumor size. At the NIH consensus conference, this risk was reported to be 25% for adrenal tumors > 6 cm, 6% for tumors between 4 and 6 cm, and 2% for tumors smaller than 4 cm [7]. This discovery led to the use of tumor size as the main parameter when deciding on the operation. In an Italian cohort, a 4-cm cutoff point was associated with 93% sensitivity and 24% specificity in preoperative diagnosis [18]. Barnett et al. identified 117 incidental adrenal masses and five tumors smaller than 5 cm out of 38 carcinomas [19]. In addition, a 4.75 cm cutoff with a sensitivity of 90% and a specificity of 58% was reported in a Korean population [20]. A recent study based in Poland found out that malignancy risk was %37.7 in tumors larger than 6 cm [21]. As is the case in the present study, in most studies, the size of the adrenal mass is considered an important criterion in differentiating between benign masses and malignant masses, but the ideal cutoff has yet to be defined [22].

There are major obstacles in the determination of an ideal cutoff value. First, the incidence of adrenal cancer in the general population is rare, and in most studies, the sample size used is insufficient, meaning such studies are inadequate to confirm the ideal cutoff [9,23]. Second, most studies collect data on cases of adrenal cancer only from surgical patients, and a control group is not available [3,17,24]. Finally, most of the data used at the NIH consensus conference was obtained from data gathered using traditional information technologies that have a lower resolution than modern technologies [7].

The incidence of adrenal adenomas increases with age but the correlation between patient age and the size of the adrenal mass is not explored in literature [25]. In our cohort, we did not find any correlation between patient age and the size of adrenal mass (*r *= 0.033).

Pathological examination revealed five (%6.57) patients had biochemically silent pheochromocytoma. One of the patients in this group had a hypertensive episode intraoperatively and had to be administered phentolamine and nitroglycerine to achieve normal blood pressure.

Regrettably, most of our patient files did not have data on metabolic parameters such as preoperative glucose tolerance, visceral fat mass, and insulin resistance, and thus we are unable to compare our patients to the normal population in this regard. Previous studies on NFAIs demonstrated an increased risk of metabolic syndrome, increased rates of impaired glucose tolerance and insulin resistance in patients with “nonfunctioning” AIs [26-28]. These metabolic disturbances altogether contribute to the cardiovascular pathologies regardless of the functional status of the mass and must be kept in mind when deciding for surgery [29].

## Conclusions

Determining the ideal cutoff value for surgical indication in a NFAI is challenging. Besides the malignancy risk, the rate of silent pheochromacytomas and metabolic burden associated with NFAIs must be taken into account in the surgical decision.
